# Intraocular hemorrhage in patients misdiagnosed with central retinal artery occlusion treated with thrombolysis

**DOI:** 10.3389/fneur.2025.1631546

**Published:** 2025-08-20

**Authors:** Daniel V. Adamkiewicz, Christian Leal, Kevin Y. Yan, Sruthi Arepalli, Kevin Ferenchak, Blaine Cribbs, Riley J. Lyons, Étienne Bénard-Séguin, Nancy J. Newman, Valérie Biousse

**Affiliations:** ^1^Department of Ophthalmology, Emory University School of Medicine, Atlanta, GA, United States; ^2^Department of Surgery, Section of Ophthalmology, University of Calgary, Calgary, AB, Canada; ^3^Department of Clinical Neurosciences, University of Calgary, Calgary, AB, Canada; ^4^Department of Neurology, Emory University School of Medicine, Atlanta, GA, United States; ^5^Department of Neurological Surgery, Emory University School of Medicine, Atlanta, GA, United States

**Keywords:** stroke, thrombolysis, retinal artery occlusion, ocular hemorrhage, case report

## Abstract

**Introduction:**

The diagnosis of acute central retinal artery occlusion (CRAO) is commonly delayed in emergency departments (ED) where ophthalmologists are rarely available for immediate consultation. Thrombolysis is sometimes given empirically for presumed CRAO without confirmation of the diagnosis with ocular funduscopic examination.

**Methods:**

We describe one case of severe intraocular hemorrhage following intravenous thrombolysis for a retinal detachment misdiagnosed as a CRAO, and two cases of worsening intraocular hemorrhage following intravenous thrombolysis for misdiagnosed CRAO, and review the literature.

**Results:**

We identified 4 cases in the literature were thrombolysis given for RAO resulted in ocular hemorrhage. We identified 12 additional cases where thrombolysis given for any indication resulted in intraocular hemorrhage.

**Discussion:**

Ocular hemorrhage is a rare but potentially devastating complication of thrombolysis in patients with underlying retinal disorders other than CRAO. Thrombolysis should never be given for acute vision loss without a funduscopic examination or ocular imaging confirming the diagnosis of CRAO.

## 1 Introduction

Central retinal artery occlusion (CRAO) is a devastating retinal infarction with very poor visual outcomes (1) and potentially life-threatening consequences (2). While no clinical trial has yet proved thrombolysis for CRAO to be efficacious, recent meta-analyses have suggested that early thrombolysis within 4.5 h of vision loss may improve visual outcomes (3, 4). As such, thrombolysis for acute CRAO is often considered a reasonable off-label hyperacute treatment when no contraindications to thrombolysis exist (2, 5, 6).

However, rapid diagnosis of CRAO in the emergency department (ED) is challenging. Delays in time to presentation and recognition of a visual complaint as a CRAO, as well as lack of availability of ophthalmologists in most general EDs, all contribute to delays in treatment. Recognizing these barriers, there has been an effort to develop reliable diagnostic modalities and treatment protocols for early detection of CRAO. Protocols using ED-based non-mydriatic optical coherence tomography (OCT) and color fundus photography have shown promising results in the rapid diagnosis of CRAO or alternate causes of vision loss (6–11). Such cameras can easily be implemented in general EDs where ocular imaging is obtained by ED staff without pharmacologic dilation of the pupils and is read remotely by an ophthalmologist, accelerating diagnosis and allowing for early management in the ED (10). Alternatively, in order to facilitate rapid management of ED patients with acute vision loss prior to stroke workup, point of care ocular ultrasound (POCUS) (12, 13) is often used by ED providers to rule-out vitreous hemorrhage and retinal detachment when no ophthalmologist is available for immediate consultation. When these common causes of vision loss are ruled-out by POCUS in the ED, a CRAO is often presumed, sometimes with subsequent empiric thrombolysis treatment without ophthalmologic examination.

Intraocular hemorrhage is a rarely reported but devastating complication of intravenous (IV) or intra-arterial (IA) thrombolysis. We describe one case of severe intraocular hemorrhage following intravenous thrombolysis for a retinal detachment misdiagnosed as a CRAO, and two cases of worsening intraocular hemorrhage following intravenous thrombolysis for misdiagnosed CRAO in the ED. We also review the existing literature reporting intraocular hemorrhage after thrombolysis.

## 2 Materials and methods

This is a case series and scoping review of intraocular hemorrhage after thrombolysis. We report 3 cases referred to our center between March 2024 and October 2024. Permission for publication of de-identified clinical information of ocular and brain imaging pictures was obtained from the patients.

A PubMed literature search was performed using different combinations of the following keywords: “central retinal artery occlusion,” “emergency department,” “acute vision loss,” “thrombolysis,” “ocular hemorrhage,” “eye stroke protocol.” The search was limited to research articles and reviews without time limits. In addition, websites of leading regulatory/health agencies were screened for relevant guidelines, and position statements. Non-English articles were excluded. We also reviewed published safety data for major thrombolysis trials in myocardial infarction (MI), pulmonary embolism (PE), and brain stroke to assess for reporting of ocular complications.

### 2.1 Case 1

A 65-year-old woman with history of hyperlipidemia, colon cancer, migraine headaches, and remote prior cataract surgery presented to an outside hospital ED with sudden painless vision loss in her left eye. Pupillary examination was documented to be unremarkable and POCUS at bedside performed to rule-out a retinal detachment or vitreous hemorrhage was unremarkable by report. The report also did not mention the presence of a “spot sign” at the optic nerve head. Funduscopic examination was attempted without pharmacologic dilation of the pupils but was unsuccessful. Computed tomography (CT) scan of the brain was normal. The diagnosis of presumed acute CRAO of the left eye was made and she was empirically treated with a bolus dose of 5.1 mg IV alteplase followed by 45.9 mg infusion over 60 min approximately 4.5 h after symptom onset. She presented to our ED 2 days later with worsening vision in the left eye. Visual acuity (VA) in the left eye was hand motion and intraocular pressure was 13 mmHg. Funduscopic examination demonstrated a dense vitreous hemorrhage and a large retinal tear and retinal detachment, which was treated with laser. At 2-month follow-up, her VA had improved to 20/30, with spontaneous improvement of vitreous hemorrhage. At 8-month follow-up, VA was 20/25, with resolution of the vitreous hemorrhage. This patient had experienced acute vision loss secondary to a retinal detachment from a large retinal tear that was complicated by vitreous hemorrhage after receiving IV thrombolysis, resulting in worsening of vision. She did not have a CRAO as the cause of her initial vision loss.

### 2.2 Case 2

**Figure 1:**
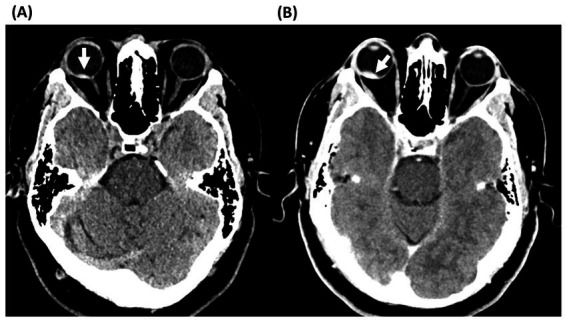
Axial head computed tomography without contrast of case 2. **(A)** CT head without contrast at initial presentation showing spontaneous intraretinal hemorrhage in the posterior pole of the right eye. **(B)** CT head after administration of intravenous thrombolysis showing enlargement of the hyperdensity, consistent with worsening of the bleed.

**Figure 2:**
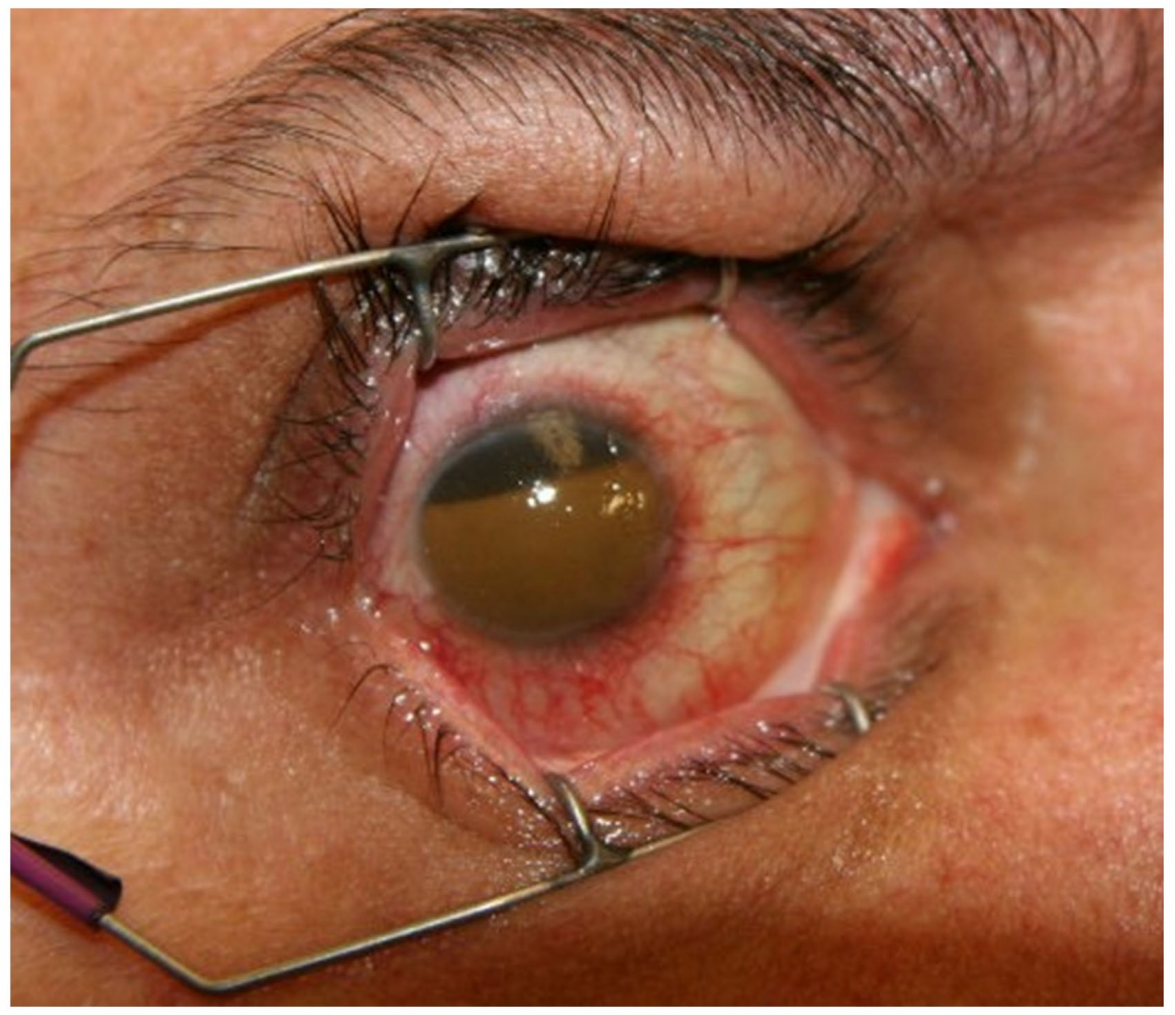
External photograph of case 2. External photograph of the right eye at two-month follow-up showing 80% filling of the anterior chamber with blood products, consistent with hemolytic glaucoma.

A 51-year-old healthy man presented to an outside hospital ED within 1 hour of sudden onset painless vision loss of his right eye. The patient endorsed heavy lifting prior to the onset of blurred vision. A head-CT was read as normal (Figure 1A). To our knowledge, no POCUS was performed, and the patient does not recall undergoing funduscopic examination in the ED. He was treated for presumed CRAO with 23.5 mg of IV tenecteplase (TNK) within 2 h of symptom onset. Five minutes later, he developed severe right eye pain. Intraocular pressure was very elevated at 84 mmHg, and head-CT scan after TNK administration was interpreted as showing a retinal detachment (Figure 1B). The patient was treated with pressure lowering drops, cryoprecipitate, and he was transferred urgently to our ED where vision in the right eye was light perception and intraocular pressure remained elevated at 48 mmHg. There was dense blood in both the anterior chamber and vitreous with poor view to the posterior pole (Figure 2). Ocular ultrasound showed dense vitreous hemorrhage, pre-hyaloid hemorrhage, and possible subretinal hemorrhage, but no retinal detachment. Review of the pre-TNK head-CT by our team showed a focal hyperdensity in the right retina suggesting a spontaneous macular hemorrhage responsible for the initial vision loss. The patient subsequently underwent retinal fluorescein angiography which showed vascular leakage along the superior vasculature in the right eye suggesting possible retinal vasculitis. Extensive laboratory and imaging workups were unremarkable. He underwent two ocular surgeries, including a combined vitrectomy and lensectomy to try to remove most of the intraocular blood, which still persisted, as did fibrotic membranes causing vitreoretinal traction. His VA in the right eye at 6-month follow-up was 20/250. The visual prognosis remains poor. He did not have a CRAO as the cause of his initial vision loss.

### 2.3 Case 3

**Figure 3:**
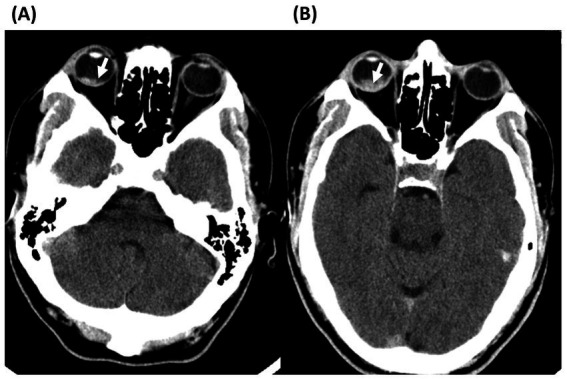
Axial head computed tomography without contrast of case 3. **(A)** CT head without contrast at initial presentation showing a spontaneous intraocular hemorrhage in the posterior pole of the right eye. **(B)** CT head after administration of intravenous thrombolysis with expansion of the hemorrhage.

A 28-year-old man with past-history of right eye trauma 1 year prior presented to an outside hospital ED with 2 h of sudden onset right eye vision loss and right-sided weakness. The patient described vision in his right eye as appearing “red.” Initial examination was notable for mild right upper and lower extremity weakness, and right eye sluggish pupillary response. Head-CT was concerning for right intraocular hemorrhage but was otherwise unremarkable (Figure 3A). Despite this finding, the patient received an 8.2 mg IV bolus of alteplase followed by a 74 mg alteplase infusion for presumed acute cerebral infarction within 4 h of symptom onset without ophthalmologic examination. His vision immediately worsened, and head-CT performed 120 min after alteplase IVT showed expanding intraocular hemorrhage (Figure 3B). He was then evaluated by the on-call ophthalmologist who noted right eye vision as light perception and intraocular pressure elevated at 34 mmHg. Funduscopic examination revealed a dense vitreous hemorrhage. He was transferred to our ED where funduscopic examination and ocular ultrasound suggested that the temporal retina might be detached in addition to vitreous hemorrhage. Magnetic resonance imaging and angiography (MRI/MRA) head and neck were normal with no evidence of acute cerebral ischemia and the diagnosis of cerebral ischemia was not confirmed by our stroke team. 3 days after initial presentation his vision in the right eye had worsened to barely light perception. Ophthalmic ultrasound demonstrated worsening of the intraocular hemorrhage. A vitrectomy was performed with intraoperative findings of five large, bleeding retinal macroaneurysms and subretinal blood through the macula and inferior retina. No retinal tears were identified. Vision 1 week after surgery remained light perception only. He did not have a CRAO as the cause of his initial vision loss.

### 2.4 Statement of ethics

This study involving human participants was exempt from the Institutional Review Board by our institution. The patients/participants provided their written informed consent to participate in this study. Permission for publication of de-identified clinical information of ocular and brain imaging pictures was obtained from the patients.

## 3 Results

Herein we report three cases of severe new intraocular hemorrhage or worsening of intraocular hemorrhage after receiving IV-thrombolysis for presumed acute CRAO. All patients had been misdiagnosed in general EDs, one despite POCUS, and should not have received thrombolysis without a funduscopic examination. In one case, subsequent hemorrhage was thought to have resulted from a pre-existing retinal tear with retinal detachment; the other two cases had-acute vision loss related to spontaneous focal retinal hemorrhages likely as a result of retinal vasculitis and bleeding macroaneurysms, respectively, and experienced dramatic worsening of their intraocular hemorrhages shortly after IV thrombolysis. Two patients received IV alteplase, and one received IV tenecteplase, all according to standard protocols for administration of IV thrombolysis for stroke within 4.5 h of vision loss. All three patients required at least one surgical intervention, and two required vitrectomies to address the intraocular hemorrhage. One patient recovered good VA, while 2 continue to have profound vision loss in the affected eye months after the initial injury.

We identified 4 previous cases in the literature in which thrombolysis given for retinal artery occlusion (RAO) resulted in intraocular hemorrhage (Table 1) (14–16). Three of these cases received intra-arterial thrombolysis and one received IV thrombolysis. None of the patients who were treated for RAO had a reported ocular condition other than the occlusion. Reported complications included pre-retinal hemorrhage (one case) (15), and other unspecified intraocular hemorrhages (two cases) (14, 16). Final VA was reported for only 1 patient and was 20/20 (branch RAO) (15).

**Table 1:** Summary of publications describing intraocular hemorrhage following thrombolysis.

Author	Year	Indication	Medication	Delivery	Patients	Ocular history	Complication	VA	Final VA
Cahane (17)	1990	MI	SK	IV	1	Recent ECCE OS	Hyphema OS	HM	20/30
Glikson (18)	1991	MI	SK	IV	1	Recent ECCE OS	Hyphema OS	NR	“no eye complaints”
Grekos (19)	1995	MI	tPA	IV	1	None	Vitreous hemorrhage OU	NR	PPV; 20/40 OU
Mahaffey (20)	1997	MI	SK	IV	1	NR	Subretinal hemorrhage OD	NR	NR
Chorich (21)	1998	MI	tPA	IV	1	Myopia	Suprachoroidal hemorrhage OD	HM	Iridectomy; LP OD
Berry (22)	2002	MI	rpA	IV	1	Wet AMD	Vitreous hemorrhage OD	LP	PPV; 4/60
Djalilian (23)	2003	MI	tPA	IV	1	Wet AMD	Vitreous hemorrhage OD	LP	LP
Kaba (24)	2005	MI	rpA	IV	1	NR	Subretinal hemorrhage OS	6/60	HM
Dhawan (25)	2014	MI	SK	IV	1	PDR	Endocapsular hematoma OS	LP	Surgery; 20/40
Shah (26)	2021	PE	tPA	IV	1	None	Choroidal/vitreous hemorrhage OD	NR	NR
Hormese (27)	2012	CVA	tPA	IV	1	NR	Vitreous hemorrhage OD	NR	LP
Shah (28)	2014	CVA	tPA	IV	1	CRVO OD	Vitreous hemorrhage OD	LP	NR
Zhang (14)	2009	RAO	UK	IAT	2	NR	“ocular fundus hemorrhage”	NR	NR
Dalzotto (15)	2021	RAO	tPA	IAT	1	None	Pre-retina hemorrhage OS	20/20	20/20
Baumgartner (16)	2023	RAO	NR	IV	1	NR	Intraocular hemorrhage	NR	NR

NR, not reported; MI, myocardial infarction; CVA, cerebrovascular accident; RAO, retinal artery occlusion; PE, pulmonary embolus; SK, streptokinase; tPA, alteplase; rPA, reteplase; UK, urokinase; IV, intravenous; IAT, intraarterial thrombolysis; VA, visual acuity; HM, hand motion; LP, light perception; OU, both eyes; OD, right eye; OS, left eye; ECCE, extracapsular cataract extraction; AMD, age related macular degeneration; PDR, proliferative diabetic retinopathy; PPV, pars plana vitrectomy; CRVO, central retinal vein occlusion.

We also identified 12 reports in which IV thrombolysis was given for brain stroke, MI, or PE and resulted in intraocular hemorrhage (Table 1) (17–28). Of these 12 cases, 6 had underlying ocular conditions that predisposed them to bleeding (17, 18, 22–24, 28), including two patients who had recently undergone cataract surgery (17, 18), 2 who had a history of exudative age-related macular degeneration (22, 23), 1 with a history of central retinal vein occlusion (28), and 1 with proliferative diabetic retinopathy (25). Ocular complications included hyphema (two cases) (17, 18), vitreous hemorrhage (five cases) (19, 22, 23, 27, 28), subretinal hemorrhage (two cases) (20, 24), choroidal hemorrhage (one case) (21), combined vitreous and choroidal hemorrhage (one case) (26), and endocapsular hematoma (one case) (25). Four of these cases required surgical intervention (19, 21, 22, 25). Final reported VA ranged from 20/30 to light perception, and 4 of these 12 patients had a final VA of hand motion or worse (21, 23, 24, 27).

We also reviewed safety data from 31 clinical trials for thrombolysis for brain stroke, MI, and PE published between 1970 and 2024. In 16 stroke trials (29–44) enrolling a combined 9,821 patients, there were 26 reported cases of ocular complications, all from only six studies (32, 35, 40, 41, 43, 44) (Table 2). Of those 6 stroke studies reporting any ocular complications of thrombolysis, rates ranged from 0.14 to 1.4%. None of the 15 MI or PE studies we reviewed reported ocular complications associated with thrombolysis (Supplementary Tables 3, 4).

**Table 2:** Ocular adverse events in large thrombolysis trials for ischemic stroke.

Study	Year	Description	Medication	Patients	Ocular adverse event
NINDS (29)	1995	tPA for acute ischemic stroke	IV tPA	624	None reported
ECASS II (30)	1998	tPA for acute ischemic stroke	IV tPA	409	None reported
ATLANTIS (31)	1999	tPA for ischemic stroke 3–5 h after symptom onset	IV tPA	272	None reported
ECASS III (32)	2008	tPA for ischemic stroke 3–4.5 h after symptom onset	IV tPA	418	1*
EPITHET (33)	2008	tPA beyond 3 h for ischemic stroke	IV tPA	52	None reported
IST-3 (34)	2012	tPA within 6 h of ischemic stroke	IV tPA	1,515	None reported
ATTEST (35)	2015	tPA vs. TNK for acute ischemic stroke	IV tPA and IV TNK	52 and 52	1 (tPA group)*
NOR-TEST (36)	2017	tPA vs. TNK for acute ischemic stroke	IV tPA and IV TNK	551 and 549	None reported
EXTEND (37)	2018	tPA vs. TNK before thrombectomy for ischemic stroke	IV tPA and IV TNK	101 and 101	None reported
WAKE-UP (38)	2018	MRI-guided thrombolysis for stroke with unknown onset	IV tPA	254	None reported
ECASS IV (39)	2019	Extending the time for thrombolysis with tPA	IV tPA	60	None reported
TRACE (40)	2021	Safety and efficacy of TNK vs. tPA in acute ischaemic stroke	IV tPA and IV TNK	59 and 181	1 (TNK 0.32 mg/kg)*
TRACE II (41)	2023	TNK vs. tPA in acute ischemic stroke	IV tPA and IV TNK	714 and 716	2 (TNK group)*
ORIGINAL (42)	2024	TNK vs. tPA in acute ischemic stroke	IV tPA and IV TNK	733 and 732	None reported
TRACE III (43)	2024	TNK for stroke at 4.5–24 h without Thrombectomy	IV TNK	264	1*
RAISE (44)	2024	Reteplase versus Alteplase for Acute Ischemic Stroke	IV tPA and IV rPA	705 and 707	8 (tPA)*, 12 (rPA)*

tPA, alteplase; TNK, tenecteplase; rPA, reteplase.

*No further detail provided regarding ocular complication.

## 4 Discussion

CRAO is a vision-threatening and time-sensitive diagnosis warranting emergent treatment prior to irreversible retinal infarction. However, many EDs lack the tools or personnel to promptly recognize this condition (8). We report three patients with painless monocular vision loss misdiagnosed as CRAO who were inappropriately treated with intravenous thrombolysis, with resultant worsening of vision loss from intraocular hemorrhage. In one case, POCUS was used in the ED prior to initiation of thrombolysis and was interpreted as normal. This patient was subsequently found to have a retinal detachment from a retinal tear. While ocular POCUS can be useful in the diagnosis of large retinal detachments and severe intraocular hemorrhages, it is not an adequate substitute for examination of the ocular fundus, as it is not sensitive enough to detect most ocular pathology when performed in the ED by ED providers using a non-dedicated ophthalmic ultrasound machine (12, 13). Additionally, although it has been proposed that ocular and orbital ultrasound can sometimes facilitate the diagnosis of CRAO by demonstrating the so-called “spot sign” (a hyperechoic signal at the level of the optic nerve suggestive of an embolic occlusion of the central retinal artery), it does not provide a definite diagnosis of CRAO and cannot replace direct visualization of the retina by funduscopic examination or ocular imaging, the latter to include color fundus photography and optical coherence tomography of the retina (12, 13, 45).

Intraocular hemorrhage is a rare but potentially devastating complication of thrombolysis (Table 1). We identified four cases in the literature who developed intraocular hemorrhage after treatment of a retinal arterial occlusion with thrombolysis (14–16) (Table 1). One patient received intra-arterial thrombolysis 9.5 h from last known normal, but the timing of thrombolysis was not available for the other three patients. As no prior ocular history was reported in 3 of these patients, it remains unknown if they had an underlying retinal condition that may have predisposed them to intraocular bleeding. We identified an additional 12 cases in the literature in which thrombolysis was given for MI (17–25), PE (26), and brain stroke (27, 28) (Table 1). Six of these cases had underlying ocular conditions that bled (17, 18, 22, 23, 25, 28) and 2 had no relevant prior ocular history (19, 26), while for three patients, ocular history was not provided (20, 24, 27).

Our three patients had underlying conditions that predisposed them to intraocular hemorrhage or worsening of intraocular hemorrhage after thrombolysis, and none had a CRAO. These patients presented to a general ED with acute monocular vision loss and were diagnosed with presumed CRAO without ocular examination. While the rate of ocular hemorrhage following thrombolysis is low in the general population, as evidenced by the paucity of reported cases in the literature and from thrombolysis trial safety data (Tables 1, 2 and Supplementary Tables 3, 4), we suspect that patients who present with acute vision loss unrelated to CRAO are at much higher risk. Indeed, these patients are far more likely to have underlying ocular pathologies predisposing them to bleeding after thrombolysis or may even have had a spontaneous intraocular bleed as the initial cause of the vision loss, as illustrated by 2 of our 3 patients. Vision loss in these cases can be profound and irreversible.

Thrombolysis for diagnosed CRAO is generally safe, with very few complications reported and complication rates similar to those of thrombolysis for MI or PE, with a very low risk of intracranial and intraocular hemorrhage (1, 2, 5). Consequently, similarly to the empiric administration of thrombolysis to patients with acute chest pain before a definite coronary syndrome is confirmed, it has been our experience that some ED providers and stroke neurologists are recommending the delivery of empiric thrombolysis for presumed CRAO in patients with acute monocular painless vision loss. Although POCUS performed in the ED can help triage patients when it demonstrates an obvious retinal detachment or vitreous hemorrhage (12, 13), it is not sensitive enough to provide a definite diagnosis of acute CRAO (45). Although we agree that patients needing systemic thrombolysis for brain stroke, MI or PE do not need an ocular examination to screen for ocular conditions that may predispose patients to ocular hemorrhages (such as proliferative diabetic retinopathy or choroidal neovascularization from age-related macular degeneration), the situation is very different when thrombolysis is considered for a patient with acute vision loss which, by definition, must have an ocular cause. There are numerous ocular causes of acute vision loss, and CRAO is relatively rare compared with other causes, including retinal detachment and vitreous or retinal hemorrhage.

A limitation of our report is that many of the thrombolysis trials we reviewed were not protocoled to report safety data on ocular complications specifically, and it is possible that the rate of intraocular hemorrhage is underreported in these trials. However, we assume that any new vision loss would have prompted further investigation in patients included in a thrombolysis clinical trial in which patient safety is always a major concern. Similarly, there are no published series of patients treated empirically with thrombolysis for presumed CRAO, which would be the only way to address the ocular safety of such treatment in the patient population with acute vision loss of any cause; however, reports of complications are rare, and we suspect that empiric administration of thrombolysis for unconfirmed CRAO is not rare.

As illustrated by our patients, administering thrombolysis to a patient with intraocular hemorrhage or retinal detachment may worsen the patient’s visual outcome and delay necessary surgical treatments. One of our patients underwent POCUS in the ED which missed a retinal tear and retinal detachment, and two of the head CTs performed in the ED as part of a stroke work up demonstrated a spontaneous intraocular hyperdensity consistent with hemorrhage that was missed in one patient and, although reported by the radiologist in the other, was either ignored or its relevance was misunderstood by the ED provider, re-enforcing that examination of the ocular fundus is essential when evaluating patients with vision loss. The lack of ophthalmic skills of most ED providers and even neurologists is well known and understandable (46), suggesting that alternative strategies in EDs receiving ocular emergencies should be considered when emergent ophthalmologic consultations are not feasible, such as implementation of non-mydriatic ocular imaging with immediate on-site interpretation or via tele-ophthalmology (7, 10, 47).

In conclusion, a thoughtful approach is required when managing acute painless monocular vision loss in the ED, balancing potential benefits of early treatment of presumed CRAO with risk of complications. As access to emergency evaluation by ophthalmologists becomes more limited at the same time stroke centers are becoming more ubiquitous and clinical trials evaluating the use of thrombolysis for acute CRAO are conducted, neurologists may feel pressured to administer thrombolysis to all potential CRAO patients presenting early enough to be eligible for this treatment. If administered for a non-ischemic ocular etiology, however, devastating intraocular complications can arise. Thrombolysis for suspected CRAO should never be given prior to examination of the ocular fundus, either by an ophthalmologist in-person, or with immediate on-site or remote interpretation of ocular imaging obtained in the ED in order to expedite the diagnosis (6–9).

## Data Availability

The datasets presented in this article are not readily available because of ethical and privacy restrictions. Requests to access the datasets should be directed to the corresponding author.

## References

[1] Kovach JL Bailey ST Kim SJ Lim JI Vemulakonda GA Ying G-S. Retinal and ophthalmic artery occlusions preferred practice pattern. Ophthalmology. (2020) 127:259–87. doi: 10.1016/j.ophtha.2024.12.024 39918522

[2] Mac Grory B Schrag M Biousse V Furie KL Gerhard-Herman M Lavin PJ. Management of central retinal artery occlusion: a scientific statement from the American Heart Association. Stroke. (2021) 52:282–94. doi: 10.1161/STR.0000000000000366, PMID: 33677974

[3] Schrag M Youn T Schindler J Kirshner H Greer D. Intravenous fibrinolytic therapy in central retinal artery occlusion: a patient-level meta-analysis. JAMA Neurol. (2015) 72:1148–54. doi: 10.1001/jamaneurol.2015.1578, PMID: 26258861

[4] Dumitrascu OM Newman NJ Biousse V. Thrombolysis for central retinal artery occlusion in 2020: time is vision! J Neuroophthalmol. (2020) 40:333–45. doi: 10.1097/WNO.0000000000001027, PMID: 32739995 PMC9548061

[5] Shah R Zheng X Patel AP Bhatti MT Gilbert A Vora RA. Central retinal artery occlusion: visual outcomes from a large northern California cohort. Ophthalmol Retina. (2024) 8:566–70. doi: 10.1016/j.oret.2023.12.007, PMID: 38154618

[6] Lema GMC De Leacy R Fara MG Ginsburg RG Barash A Banashefski B. A remote consult retinal artery occlusion diagnostic protocol. Ophthalmology. (2024) 131:724–30. doi: 10.1016/j.ophtha.2023.11.031, PMID: 38349294

[7] Bénard-Séguin É Nahab F Pendley AM duran MR Soto MT Keadey M. Eye stroke protocol in the emergency department. J Stroke Cerebrovasc Dis. (2024) 33:107895. doi: 10.1016/j.jstrokecerebrovasdis.2024.107895 39079617

[8] Dumitrascu OM English S Alhayek N Pahl E Nord C Vanderhye V. Telemedicine for acute monocular visual loss: a retrospective large telestroke network experience. Telemed J E Health. (2023) 29:1738–43. doi: 10.1089/tmj.2022.0286, PMID: 36912816

[9] Gilbert AL Patel AP Sax D Bhatti MT Shah R Dokey A. A telemedicine-enabled intravenous thrombolytic treatment pathway for patients with hyperacute non-arteritic central retinal artery occlusion. Am J Ophthalmol Case Rep. (2024) 36:102204. doi: 10.1016/j.ajoc.2024.102204, PMID: 39512749 PMC11541670

[10] Berman G Pendley AM Wright DW Silverman R Kelley C Duran MR. Breaking the barriers: methodology of implementation of a non-mydriatic ocular fundus camera in an emergency department. Surv Ophthalmol. (2025) 70:153–61. doi: 10.1016/j.survophthal.2024.09.012 39357747

[11] Shanmugam N Benard-Seguin E Arepalli S Alencastro G McHenry JG Rodriguez Duran M. Remote diagnosis of retinal detachment in an emergency department using nonmydriatic hybrid ocular imaging. Telemed J E Health. (2025) 31:185–90. doi: 10.1089/tmj.2024.0435, PMID: 39347598

[12] Skidmore C Saurey T Ferre RM Rodriguez-Brizuela R Spaulding J Lundgreen Mason N. A narrative review of common uses of ophthalmic ultrasound in emergency medicine. J Emerg Med. (2021) 60:80–9. doi: 10.1016/j.jemermed.2020.08.003, PMID: 32919837

[13] Schott ML Pierog JE Williams SR. Pitfalls in the use of ocular ultrasound for evaluation of acute vision loss. J Emerg Med. (2013) 44:1136–9. doi: 10.1016/j.jemermed.2012.11.079, PMID: 23522956

[14] Zhang X Ji X Luo Y Liu D Guo L Wu H. Intra-arterial thrombolysis for acute central retinal artery occlusion. Neurol Res. (2009) 31:385–9. doi: 10.1179/174313209X444008, PMID: 19508824

[15] Dalzotto K Richards P Boulter TD Kay M Mititelu M. Complications of intra-arterial tPA for iatrogenic branch retinal artery occlusion: a case report through multimodal imaging and literature review. Medicina (Kaunas). (2021) 57:963. doi: 10.3390/medicina57090963, PMID: 34577886 PMC8464858

[16] Baumgartner P Kook L Altersberger VL Gensicke H Ardila-Jurado E Kägi G. Safety and effectiveness of IV thrombolysis in retinal artery occlusion: a multicenter retrospective cohort study. Eur Stroke J. (2023) 8:966–73. doi: 10.1177/23969873231185895, PMID: 37421135 PMC10683723

[17] Cahane M Ashkenazi I Avni I Blumenthal M. Total hyphema following streptokinase administration eight days after cataract extraction. Br J Ophthalmol. (1990) 74:447. doi: 10.1136/bjo.74.7.447-a, PMID: 2378862 PMC1042163

[18] Glikson M Feinberg M Hod H Kaplinsky E. Thrombolytic therapy for acute myocardial infarction following recent cataract surgery. Am Heart J. (1991) 121:1542–3. doi: 10.1016/0002-8703(91)90164-D, PMID: 2017986

[19] Grekos ZG Schocken DD. Bilateral vitreous hemorrhages as a consequence of thrombolytic therapy successfully treated with vitrectomy in a patient without diabetes. Am Heart J. (1995) 130:611–2. doi: 10.1016/0002-8703(95)90371-2 7661080

[20] Mahaffey KW Granger CB Toth CA White HD Stebbins AL Barbash GI. Diabetic retinopathy should not be a contraindication to thrombolytic therapy for acute myocardial infarction: review of ocular hemorrhage incidence and location in the GUSTO-I trial. Global utilization of streptokinase and t-PA for occluded coronary arteries. J Am Coll Cardiol. (1997) 30:1606–10. doi: 10.1016/s0735-1097(97)00394-x, PMID: 9385883

[21] Chorich LJ Derick RJ Chambers RB Cahill KV Quartetti EJ Fry JA. Hemorrhagic ocular complications associated with the use of systemic thrombolytic agents. Ophthalmology. (1998) 105:428–31. doi: 10.1016/S0161-6420(98)93023-8, PMID: 9499772

[22] Berry C Weir C Hammer H. A case of intraocular haemorrhage secondary to thrombolytic therapy. Acta Ophthalmol Scand. (2002) 80:561–2. doi: 10.1034/j.1600-0420.2002.800522_1.x, PMID: 12390175

[23] Djalilian AR Cantrill HC Samuelson TW. Intraocular hemorrhage after systemic thrombolytic therapy in a patient with exudative macular degeneration. Eur J Ophthalmol. (2003) 13:96–8. doi: 10.1177/112067210301300117, PMID: 12635684

[24] Kaba RA Cox D Lewis A Kaba R Bloom P Dubrey S. Intraocular haemorrhage after thrombolysis. Lancet. (2005) 365:330–08. doi: 10.1016/S0140-6736(05)70198-7, PMID: 15664229

[25] Dhawan B Soni R Singh R Vig V. Endocapsular hematoma: a rare form of ocular hemorrhage after thrombolysis with streptokinase. N Am J Med Sci. (2014) 6:425–7. doi: 10.4103/1947-2714.139310, PMID: 25210679 PMC4158654

[26] Shah TE Vij R Kim YH Shiloh AL. Sudden unilateral vision loss in a patient who received intravenous thrombolytic therapy. Chest. (2021) 160:e669–72. doi: 10.1016/j.chest.2021.03.074, PMID: 34872684

[27] Hormese M Wichter M. Vitreo-retinal hemorrhage after thrombolysis in a patient with acute ischemic stroke: a case report. Front Neurol. (2012) 3:71. doi: 10.3389/fneur.2012.00071, PMID: 22586418 PMC3347041

[28] Shah L Verstraeten T Wright D Rana S. Vitreous hemorrhage as a complication of IV-tPA therapy in a patient with acute stroke. Neurology. (2014) 82:227. doi: 10.1212/WNL.82.10_supplement.P4.227

[29] National Institute of Neurological Disorders and Stroke rt-PA Stroke Study Group. Tissue plasminogen activator for acute ischemic stroke. N Engl J Med. (1995) 333:1581–7. 7477192 10.1056/NEJM199512143332401

[30] Hacke W Kaste M Fieschi C von Kummer R Davalos A Meier D. Randomised double-blind placebo-controlled trial of thrombolytic therapy with intravenous alteplase in acute ischaemic stroke (ECASS II). Lancet. (1998) 352:1245–51. doi: 10.1016/S0140-6736(98)08020-9, PMID: 9788453

[31] Clark WM Wissman S Albers GW Jhamandas JH Madden KP Hamilton S. Recombinant tissue-type plasminogen activator (alteplase) for ischemic stroke 3 to 5 hours after symptom onset. The ATLANTIS study: a randomized controlled trial. JAMA. (1999) 282:2019–26. 10591384 10.1001/jama.282.21.2019

[32] Hacke W Kaste M Bluhmki E Brozman M Dávalos A Guidetti D. Thrombolysis with alteplase 3 to 4.5 hours after acute ischemic stroke. N Engl J Med. (2008) 359:1317–29. doi: 10.1056/NEJMoa0804656, PMID: 18815396

[33] Davis SM Donnan GA Parsons MW Levi C Butcher KS Peeters A. Effects of alteplase beyond 3 h after stroke in the Echoplanar imaging thrombolytic evaluation trial (EPITHET): a placebo-controlled randomised trial. Lancet Neurol. (2008) 7:299–309. doi: 10.1016/S1474-4422(08)70044-9, PMID: 18296121

[34] Sandercock P Wardlaw JM Lindley RI Dennis M Cohen G Murray G. The benefits and harms of intravenous thrombolysis with recombinant tissue plasminogen activator within 6 h of acute ischaemic stroke (the third international stroke trial [IST-3]): a randomised controlled trial. Lancet. (2012) 379:2352–63. doi: 10.1016/S0140-6736(12)60768-5, PMID: 22632908 PMC3386495

[35] Huang X Cheripelli BK Lloyd SM Kalladka D Moreton FC Siddiqui A. Alteplase versus tenecteplase for thrombolysis after ischaemic stroke (ATTEST): a phase 2, randomised, open-label, blinded endpoint study. Lancet Neurol. (2015) 14:368–76. doi: 10.1016/S1474-4422(15)70017-7, PMID: 25726502

[36] Logallo N Novotny V Assmus J Kvistad CE Alteheld L Rønning OM. Tenecteplase versus alteplase for management of acute ischaemic stroke (NOR-TEST): a phase 3, randomised, open-label, blinded endpoint trial. Lancet Neurol. (2017) 16:781–8. doi: 10.1016/S1474-4422(17)30253-3, PMID: 28780236

[37] Campbell BCV Mitchell PJ Churilov L Yassi N Kleinig TJ Dowling RJ. Tenecteplase versus alteplase before thrombectomy for ischemic stroke. N Engl J Med. (2018) 378:1573–82. doi: 10.1056/NEJMoa1716405, PMID: 29694815

[38] Thomalla G Simonsen CZ Boutitie F Andersen G Berthezene Y Cheng B. MRI-guided thrombolysis for stroke with unknown time of onset. N Engl J Med. (2018) 379:611–22. doi: 10.1056/NEJMoa1804355, PMID: 29766770

[39] Ringleb P Bendszus M Bluhmki E Donnan G Eschenfelder C Fatar M. Extending the time window for intravenous thrombolysis in acute ischemic stroke using magnetic resonance imaging-based patient selection. Int J Stroke. (2019) 14:483–90. doi: 10.1177/1747493019840938, PMID: 30947642

[40] Li S Pan Y Wang Z Liang Z Chen H Wang D. Safety and efficacy of tenecteplase versus alteplase in patients with acute ischaemic stroke (TRACE): a multicentre, randomised, open label, blinded-endpoint (PROBE) controlled phase II study. Stroke Vasc Neurol. (2022) 7:47–53. doi: 10.1136/svn-2021-000978, PMID: 34429364 PMC8899644

[41] Wang Y Li S Pan Y Li H Parsons MW Campbell BCV. Tenecteplase versus alteplase in acute ischaemic cerebrovascular events (TRACE-2): a phase 3, multicentre, open-label, randomised controlled, non-inferiority trial. Lancet. (2023) 401:645–54. doi: 10.1016/S0140-6736(22)02600-9, PMID: 36774935

[42] Meng X Li S Dai H Lu G Wang W Che F. Tenecteplase vs alteplase for patients with acute ischemic stroke: the ORIGINAL randomized clinical trial. JAMA. (2024) 332:1437–45. doi: 10.1001/jama.2024.14721, PMID: 39264623 PMC11393753

[43] Xiong Y Campbell BCV Schwamm LH Meng X Jin A Parsons MW. Tenecteplase for ischemic stroke at 4.5 to 24 hours without thrombectomy. N Engl J Med. (2024) 391:203–12. doi: 10.1056/NEJMoa2402980, PMID: 38884324

[44] Li S Gu HQ Dai H Lu G Wang Y. Reteplase versus alteplase for acute ischaemic stroke within 4.5 hours (RAISE): rationale and design of a multicentre, prospective, randomised, open-label, blinded-endpoint, controlled phase 3 non-inferiority trial. Stroke Vasc Neurol. (2024) 9:568–73. doi: 10.1136/svn-2023-003035, PMID: 38286482 PMC11732833

[45] Taroza S Jatužis D Matijošaitis V Raugelė S Valaikienė J. Central retinal artery occlusion or retinal stroke: a neurosonologist's perspective. Front Neurol. (2024) 15:1397751. doi: 10.3389/fneur.2024.1397751, PMID: 38915799 PMC11194405

[46] Biousse V Bruce BB Newman NJ. Ophthalmoscopy in the 21st century: the 2017 H. Houston Merritt lecture. Neurology. (2018) 90:167–75. doi: 10.1212/WNL.0000000000004868, PMID: 29273687 PMC5798658

[47] English SW Barrett KM Freeman WD Demaerschalk BM Dumitrascu OM. Improving the telemedicine evaluation of patients with acute vision loss: a call to eyes. Neurology. (2022) 99:381–6. doi: 10.1212/WNL.0000000000200969, PMID: 35764399

