# Supplementary material for: Intraocular hemorrhage in patients misdiagnosed with central retinal artery occlusion treated with thrombolysis
Source: Front Neurol. 2025 Aug 20;16:1631546. doi: 10.3389/fneur.2025.1631546 (PMC12404925; doi:10.3389/fneur.2025.1631546)
Supplement: Supplementary file 1 [file Supplementary_file_1.docx]

| Supplementary Material  **Table S1. Ocular adverse events in large thrombolysis trials for myocardial infarction** | | | | |  |  |  |
| --- | --- | --- | --- | --- | --- | --- | --- |
| Study | Year | Description | Medication | Patients | | Ocular Adverse Event | |
| GISSI^1^ | 1987 | Standard of care vs SK for MI | IV SK | 5,225 | | None reported | |
| TIMI^2^ | 1987 | Thrombolysis for MI | IV tPA vs IV SK | 143 vs 147 | | None reported | |
| TAMI^3^ | 1987 | Thrombolysis and angioplasty for MI | IV tPA | 386 | | None reported | |
| ISIS 2^4^ | 1988 | IV SK, oral ASA, both, or neither for MI | IV SK | 8,592 | | None reported | |
| TIMI II^5^ | 1989 | Invasive vs conservative treatment after IV tPA for MI | IV tPA | 3,262 | | None reported | |
| ISIS 3^6^ | 1992 | SK vs tPA vs aPA with ASA + heparin vs ASA alone for MI | IV SK vs IV tPA vs IV aPA | 13,780 vs 13,746, vs 13,773 | | None reported | |
| GUSTO^7^ | 1993 | Comparison of thrombolytic strategies for MI | IV SK vs IV tPA vs IV SK+ IV tPA | 20,245 vs 10,396 vs 10,374 | | None reported | |
| TIMI IIIB^8^ | 1994 | tPA vs placebo for unstable angina | IV tPA | 729 | | None reported | |
| GUSTO IIB^9^ | 1997 | Angioplasty vs thrombolysis for MI | IV tPA | 573 | | None reported | |
| GUSTO III^10^ | 1997 | rPA vs tPA for MI | IV tPA vs IV rPA | 10,138 vs 4,921 | | None reported | |
|  |  |  |  |  | |  | |
| MI = myocardial infarction; SK = Streptokinase; tPA = Alteplase; TNK = Tenecteplase; rPA = Reteplase; aPA = Anistreplase; ASA = Aspirin | | | | | | |  |

| **Table S2. Ocular adverse events in thrombolysis trials for pulmonary embolism** | | | | |  |  |  |
| --- | --- | --- | --- | --- | --- | --- | --- |
| Study | Year | Description | Medication | Patients | | Ocular Adverse Event | |
| UPET^11^ | 1970 | Urokinase for PE | IV Urokinase | 82 | | None reported | |
| PIOPED^12^ | 1990 | Alteplase for PE | IV tPA | 13 | | None reported | |
| PAIMS2^13^ | 1992 | Alteplase vs heparin for PE | IV tPA | 20 | | None reported | |
| mOPETT^14^ | 2013 | Alteplase for moderate PE | IV tPA | 61 | | None reported | |
| PEITHO^15^ | 2014 | Tenecteplase for intermediate risk PE | IV TNK | 506 | | None reported | |
|  |  |  |  |  | |  | |
| PE = pulmonary embolism; tPA = Alteplase; TNK = Tenecteplase | | | | | | |  |

**References:**

1. Rovelli F, De Vita C, Feruglio GA, et al. GISSI trial: early results and late follow-up. Gruppo Italiano per la Sperimentazione della Streptochinasi nell'Infarto Miocardico. *J Am Coll Cardiol* 1987; 10: 33B-39B.
2. Chesebro JH, Knatterud G, Roberts R, et al. Thrombolysis in Myocardial Infarction (TIMI) Trial, Phase I: A comparison between intravenous tissue plasminogen activator and intravenous streptokinase. Clinical findings through hospital discharge. *Circulation* 1987; 76: 142-154.
3. Topol EJ, Califf RM, Kereiakes DJ, et al. Thrombolysis and Angioplasty in Myocardial Infarction (TAMI) trial. *J Am Coll Cardiol* 1987; 10: 65B-74B.
4. Randomised trial of intravenous streptokinase, oral aspirin, both, or neither among 17,187 cases of suspected acute myocardial infarction: ISIS-2. ISIS-2 (Second International Study of Infarct Survival) Collaborative Group. *Lancet* 1988; 2: 349-360.
5. Thrombolysis in myocardial infarction (TIMI) phase II trial. *N Engl J Med* 1989; 321: 612.
6. ISIS-3: a randomised comparison of streptokinase vs tissue plasminogen activator vs anistreplase and of aspirin plus heparin vs aspirin alone among 41,299 cases of suspected acute myocardial infarction. ISIS-3 (Third International Study of Infarct Survival) Collaborative Group. *Lancet* 1992; 339: 753-770.
7. GUSTO investigators. An international randomized trial comparing four thrombolytic strategies for acute myocardial infarction. *N Engl J Med* 1993; 329: 673-682.
8. Effects of tissue plasminogen activator and a comparison of early invasive and conservative strategies in unstable angina and non-Q-wave myocardial infarction. Results of the TIMI IIIB Trial. Thrombolysis in Myocardial Ischemia. *Circulation* 1994; 89: 1545-1556.
9. Global Use of Strategies to Open Occluded Coronary Arteries in Acute Coronary Syndromes (GUSTO IIb) Angioplasty Substudy Investigators. A clinical trial comparing primary coronary angioplasty with tissue plasminogen activator for acute myocardial infarction. *N Engl J Med* 1997; 336: 1621-1628.
10. Global Use of Strategies to Open Occluded Coronary Arteries (GUSTO III) Investigators. A comparison of reteplase with alteplase for acute myocardial infarction. *N Engl J Med* 1997; 337: 1118-1123.
11. Urokinase pulmonary embolism trial. Phase 1 results: a cooperative study. *JAMA* 1970; 214: 2163-2172.
12. Tissue plasminogen activator for the treatment of acute pulmonary embolism. A collaborative study by the PIOPED Investigators. *Chest* 1990; 97: 528-533.
13. Dalla-Volta S, Palla A, Santolicandro A, et al. PAIMS 2: alteplase combined with heparin versus heparin in the treatment of acute pulmonary embolism. Plasminogen activator Italian multicenter study 2. *J Am Coll Cardiol* 1992; 20: 520-526.
14. Sharifi M, Bay C, Skrocki L, et al. Moderate pulmonary embolism treated with thrombolysis (from the "MOPETT" Trial). *Am J Cardiol* 2013; 111: 273-277.
15. Meyer G, Vicaut E, Danays T, et al. Fibrinolysis for patients with intermediate-risk pulmonary embolism. *N Engl J Med* 2014; 370:1402-1411.
